# Dopamine Dysregulation Syndrome in Parkinson’s Disease Patients with Unsatisfactory Switching from Immediate to Extended Release Pramipexole: A Further Clue to Incentive Sensitization Mechanisms?

**DOI:** 10.3233/BEN-129026

**Published:** 2013

**Authors:** Paolo Solla, Antonino Cannas, Marta Corona, Maria Giovanna Marrosu, Francesco Marrosu

**Affiliations:** ^1^aMovement Disorders CenterInstitute of NeurologyUniversity of CagliariCagliariItaly; ^2^MS CenterInstitute of NeurologyUniversity of CagliariCagliariItaly

**Keywords:** Dopamine dysregulation syndrome, Parkinson’s disease, dopamine agonists, short-acting drugs

## Abstract

A small proportion of patients with Parkinson’s disease (PD), chronically under dopamine replacement therapy, may undergo an addiction-like behavioral disturbance, named dopamine dysregulation syndrome (DDS). This behavioral disorder is characterized by the increase of doses beyond those required for motor control, and its management remains difficult; thus, early recognition and careful monitoring of at-risk individuals are crucial. We report the cases of two PD patients with a previous unsatisfactory switching from an immediate release (IR) to an extended release (ER) pramipexole formulation who developed DDS. PD patients unsatisfactorily switched from an IR to an ER formulation of dopamine agonists should be considered as at-risk individuals for DDS development.

## 1. Introduction

Dopamine replacement therapy (DRT) constitutes the standard treatment for motor symptoms in patients affected by Parkinson's disease (PD). However, due to the progressive course of the neurodegenerative process, the doses of DRT required to control motor disorders increase during the evolution of the disease. A small proportion of PD patients, chronically treated with dopaminergic drugs, may undergo an addiction-like behavioral disturbance, named dopamine dysregulation syndrome (DDS) [1]. This behavioral disorder is associated with substance misuse and addiction, characterized by the increase of DRT doses continuously beyond those required for motor control, despite severe drug induced dyskinesias. Besides, patients may also develop a cyclical mood disorder with hypomania or manic psychosis [1].

Overall, DDS in PD is difficult to treat and often complicated by a lack of insight of the patient; thus, early recognition and careful monitoring of at-risk individuals are crucial.

Young age at PD onset, high novelty-seeking personality traits and psychosocial factors such as substance abuse and depression may predispose patients to de-

ISSN 0953-4180/13/$27.50 © 2013 – IOS Press and the authors. All rights reserved veloping DDS [2], which is associated with high levodopa and levodopa equivalent doses [3]. Dopaminergic drugs usually related to DDS are short-acting "rescue" medications as dispersible levodopa and intermittent injections of apomorphine [1]. Management and prevention of DDS is mainly based on the model of the continuous stimulation of dopamine receptors, with the discontinuation of "rescue" medication [1]. Consistent with this concept, the remission of DDS in a patient following a switch to continuous intraduodenal levodopa infusion has been recently described [4]. In this context, the use of long-acting dopaminergic drugs such as once-daily therapies, providing a more continuous stimulation of dopamine receptors, might theoretically provide a reduced risk for this complication.

Recently, an extended-release (ER) pramipexole formulation taken once daily, has demonstrated to provide comparable benefit resembling those of immediate-release (IR) pramipexole taken three times daily (t.i.d.) both in early and advanced PD [5,6]. However, differences on non-motor symptoms between IR and ER pramipexole formulations have not been systematically explored.

Here we describe the cases of two patients affected by Parkinson's disease with an unsatisfactory switching from an immediate release (IR) to an extended release (ER) pramipexole formulation who developed DDS.

*Case 1.* This 51-year-old male had suffered from PD for 14 years. He initially presented with mild rest tremor of the left hand, associated with mild bradykinesia. His past medical history was unremarkable and he had no personal or family history of psychiatric disorders or substance abuse. His parkinsonian symptoms progressed over the next 12 years, and dopaminergic therapy was introduced at increasing dosage (levodopa 700 mg/daily and pramipexole IR 0.7 mg q.i.d.), with subjective clinical benefit, although motor fluctuations were reported. Two years later, he began to complain of mood fluctuations, during which he experienced moderate depressive symptoms with severe anxiety. The MINI International Neuropsychiatric Interview [7] was used to investigate the presence of depressive symptoms and anxiety, while Hamilton Depression Rating Scale (17 items) [8] score was 20. Later occurrence of unpredictable off phenomena, prompted us to switch pramipexole from IR to ER formulation at 3.15 mg daily, with concomitant worsening of anxiety and depression. The patient spontaneously decided to re-switch to pramipexole IR 0.7 mg q.i.d., with better subjective perception of on state, although more severe dyskinesias and mood fluctuations were registered. After three weeks, the patient started to self-administer excessive extra doses of dopaminergic drugs, doubling levodopa doses and developing disabling drug-induced dyskinesias with rapid shifts in mood and episodic hypomanic behavioral disorder. He said he needed this enhancement of DRT doses, although in excess of those required to control his motor symptoms, in order to avoid depressive mood and severe anxiety. These features fulfilled the diagnosis of DDS [1], according to Giovannoni's criteria [1]. Questionnaire for Impulsive-Compulsive Disorders in PD [9] did not show any impulse control disorders such as pathological gambling, sex, buying, and eating disorders. Montreal Cognitive assessment (MoCA) score was 30 (of 30), and his Frontal Assessment Battery (FAB) score was 16 (of 18). Reduction of DRT was not tolerated due to worsening of both motor and depressive symptoms. Although discouraged, the patient perpetuated in drug abuse.

*Case 2.* A 74-year-old retired small businessman was diagnosed with PD at age 63. He initially presented with rest tremor of the left arm and hand, associated with mild rigidity and bradykinesia. He was an ex-smoker and his medical history included mild hypertension, while there was no family history of neurological diseases or mood disorders. He described himself as a determined, although sometimes impulsive, subject in the working activity, and had always been a novelty seeker. After eleven years of disease, he complained of motor fluctuations, such as wearing off phenomena, while mild dyskinesias were noted. At that time, he was being treated with levodopa ER 600 mg/day, rasagiline 1 mg/day, amantadine 50 mg/day and pramipexole IR 0.7 mg tid. With the aim to improve motor fluctuations, his medication was changed with an overnight switch from pramipexole IR to pramipexole ER, 2.1 mg daily. However, because the patient complained of more severe off-periods, he discontinued pramipexole ER and spontaneously re-administered pramipexole IR 0.7 mg, which was increased to five times a day. He referred that the administration of IR formulation helped him to feel better, improving anxiety and depressive symptoms. Two weeks later, he began to take more doses of levodopa ER than prescribed up to 1400 mg/day, despite an adequate motor control at lower doses. Over the next 6 months the dyskinesias became more marked and he developed marked mood fluctuations with periods of depressed mood, agitation and incapacitating anxiety alternated with periods of hypomania. Hamilton Depression Rating Scale (17 items) [8] score registered in the off-period was 41. Neurological examination revealed moderate parkinsonism, with bradykinesia and left side-dominant rest tremor in off state, and severe and disabling drug-induced dyskinesias in on periods, although he did not complain of dyskinetic movements. MoCA score was 27/30 and FAB score was 16/18. Visual hallucinations or delusional ideas were not reported. The patient fulfilled clinical criteria for DDS, based on Giovannoni's criteria [1]. Questionnaire for Impulsive-Compulsive Disorders in PD [9] did not show any impulse control disorders such as pathological gambling, sex, buying, and eating disorders.

With the aid of his relatives, we gradually reduced dopaminergic drugs, with a clear improvement in compulsive behavior, although the patient complained of severe off periods.

## 2. Discussion

We described two patients who developed DDS, subsequently to an unsuccessful switching from an immediate release (IR) to an extended release (ER) formulation of pramipexole.

PD patients with DDS represent a major management challenge and to date there are not universally successful treatments for this disorder. The main difficulty is represented by the fact that the pathophysiology of DDS is multifactorial and not completely understood. Several potential risk factors have been implicated including genetic risk factors, habit formation with negative reinforcement resulting by unpleasant off motor and non-motor symptoms, presence of mood disorders, fronto-striatal dysfunction and sensitization processes due to repeated dopaminergic stimulation [10–12]. All these factors may play a role variable from case to case.

Our patients suffered from an advanced form of disease with motor and especially severe non motor affective fluctuations associated with dyskinesias. Despite disease duration, patients did not show an evident cognitive dysfunction. Such finding in these patients with tremor-dominant PD is not unusual, while several studies have demonstrated that non-tremor-dominant PD is strongly associated with cognitive deterioration [13, 14].

The unsatisfactory switching from an IR to an ER formulation of the dopamine agonist could be explained by the different drug delivery mechanisms and the better subjective improvement of affective symptoms with IR formulation. For instance, a recent clinical trial in advanced PD patients showed a clear significant improvement in patients' global self-ratings for pramipexole IR, while was only at margin of statistical significance for ER formulation [6]. In addition, the same study reported that quality of life was significantly improved for IR but not for ER pramipexole. Indeed, pramipexole IR, differently from ER formulation, may act as a short acting drug, with pulsatile mechanism more similar to levodopa which is the typical short-acting "rescue" drug in PD. This pulsatile dopamine-receptor stimulation is postulated to induce both plastic changes in motor systems and persistent neuroadaptations induced in dopamine projections to the accumbens-related circuitry which are respectively responsible for the development of motor and non-motor long-term complication of DRT in PD [10–12].

The concept that short-acting drugs are believed to induce both motor and non-motor sensitization via pulsatile receptor stimulation constitutes the base for the theory of incentive sensitization [11]. With regard to non-motor symptoms, the circuitry of the nucleus accumbens becomes hypersensitive to the rewarding effects of repeatedly administered drugs [15].

Consistent with this hypothesis, the mechanism of pulsatile receptor stimulation explicates why standard levodopa, which has a very short half-life, has been associated to DDS. Indeed, PD patients with DDS showed enhanced levodopa-induced ventral striatal dopamine release compared with levodopa treated PD patients without this compulsive behavior [16].

Actually, the predilection in our patients for an IR formulation resulted in the clear development of a pattern of compulsive drug taking. In this context, the basis for this drug-induced pathologic behavior might be related to both alterations of brain's reward system and drug related aspects such as pulsatile dopamine receptors stimulations. According to this hypothesis, the sensitized systems mediate a subcomponent of reward termed incentive salience, which leads to the compulsive drug seeking behavior [17].

In conclusion, we retain that PD patients with unsatisfactory switching from an IR to an ER formulation of dopamine agonists should be considered as at-risk individuals for DDS development.

## References

[1] Giovannoni G, O'Sullivan JD, Turner K, Manson AJ, Lees AJ. Hedonistic homeostatic dysregulation in patients with Parkinson's disease on dopamine replacement therapies. J Neurol Neurosurg Psychiatry. 2000; 68(4): 423-428.

[2] Evans AH, Lawrence AD, Potts J, Appel S, Lees AJ. Factors influencing susceptibility to compulsive dopaminergic drug use in Parkinson disease. Neurology. 2005; 65(10): 1570-1574.

[3] Gallagher DA, O'Sullivan SS, Evans AH, Lees AJ, Schrag A. Pathological gambling in Parkinson's disease: risk factors and differences from dopamine dysregulation. An analysis of published case series. Mov Disord. 2007; 22: 1757-1763.

[4] Gerschlager W, Bloem BR. Managing pathological gambling in Parkinson's disease with enteral levodopa/carbidopa infusions. Mov Disord. 2009; 24: 1858-1860.

[5] Poewe W, Rascol O, Barone P, et al. Extended-release pramipexole in early Parkinson disease: A 33-week randomized controlled trial. Neurology. 2011; 77: 759-766.

[6] Shapira AHV, Barone P, Hauser RA, et al. Extended-release pramipexole in advanced Parkinson disease: A randomized controlled trial. Neurology. 2011; 77: 767-774.

[7] Sheehan DV, Lecrubier Y, Sheehan KH, et al. The Mini-International Neuropsychiatric Interview (M. I. N. I.): the development and validation of a structured diagnostic psychiatric interview for DSM-IV and ICD- 10. J Clin Psychiatry. 1998; 59: 34-57.

[8] Hamilton M. Development of a rating scale for primary depressive illness. Br J Soc Clin Psychol. 1967; 6(4): 278-296.

[9] Weintraub D, Hoops S, Shea JA, et al. Validation of the questionnaire for impulsive-compulsive disorders in Parkinson's disease. Mov Disord. 2009; 24(10): 1461-1467.

[10] Rascol O. Drugs and drug delivery in PD: optimizing control of symptoms with pramipexole prolonged-release. Eur J Neurol. 2011; 18 Suppl 1: 3-10.

[11] Katzenschlager R. Dopaminergic dysregulation syndrome in Parkinson's disease. J Neurol Sci. 2011; 310(1–2): 271-275.

[12] Lawrence AD, Evans AH, Lees AJ. Compulsive use of dopamine replacement therapy in Parkinson's disease: reward systems gone awry? Lancet Neurol. 2003; 2: 595-604.

[13] Selikhova M, Williams DR, Kempster PA, Holton JL, Revesz T, Lees AJ. A clinico-pathological study of subtypes in Parkinson's disease. Brain. 2009; 132(Pt 11): 2947-2957.

[14] Reijnders JS, Ehrt U, Lousberg R, Aarsland D, Leentjens AF. The association between motor subtypes and psychopathology in Parkinson's disease. Parkinsonism Relat Disord. 2009; 15(5): 379-382.

[15] Evans AH, Lawrence AD, Cresswell SA, Katzenschlager R, Lees AJ. Compulsive use of dopaminergic drug therapy in Parkinson's disease: reward and anti-reward. Mov Disord. 2010; 25: 867–876.

[16] Evans, AH, Pavese N, Lawrence AD, Tai YF, Appel S, Doder M, Brooks DJ, Lees AJ, Piccini P. Compulsive Drug Use Linked to Sensitized Ventral Striatal Dopamine Transmission Ann Neurol. 2006; 59: 852-858.

[17] Berridge KC, Robinson TE. What is the role of dopamine in reward: hedonic impact, reward learning, or incentive salience? Brain Res Revs. 1998; 28: 309-369.
